# Supplementary material for: Identification and assessment of a comprehensive set of structural factors associated with hospital costs in Switzerland
Source: PLoS One. 2022 Feb 17;17(2):e0264212. doi: 10.1371/journal.pone.0264212 (PMC8853497; doi:10.1371/journal.pone.0264212)
Supplement: S1 Table — (DOCX) [file pone.0264212.s001.docx]

**S1 Table** *Descriptive statistics (means, standard deviations (SD), and ranges) of all investigated variables*

|  | **University** | **Central** | **Regional** | **Birth** | **Children** | **Specialty** | **All Hospitals** |
| --- | --- | --- | --- | --- | --- | --- | --- |
| **V1 - Number of beds** | |  |  |  |  |  |  |
| ***Mean (SD)*** | 1,143.64 (449.46) | 344.09 (197.12) | 86.39 (54.12) | 3.61 (1.92) | 136.32 (66.84) | 62.91 (62.88) | 203.06 (276.71) |
| ***Range*** | 663.0-1,828.73 | 129.56-938.0 | 7.0-254.47 | 1.07-7.34 | 82.0-210.96 | 13.09-301.0 | 1.07-1,828.73 |
| **V2 - Number of discharges** | |  |  |  |  |  |  |
| ***Mean (SD)*** | 46,544.4 (6,921.79) | 18,256.05 (9,793.9) | 4,867.88 (2,691.18) | 376.5 (286.61) | 6,275.33 (2,205.67) | 2,815.18 (2,103.96) | 10,172.77 (11,854.06) |
| ***Range*** | 37,503.0-56,157.0 | 3,177.0-42,706.0 | 406.0-9,629.0 | 110.0-892.0 | 4,162.0-8,563.0 | 153.0-7,798.0 | 110.0-56,157.0 |
| **V3 - Number of departments** | |  |  |  |  |  |  |
| ***Mean (SD)*** | 11.6 (1.14) | 6.76 (1.92) | 4.88 (1.72) | 1.0 (0.0) | 4.33 (4.16) | 2.91 (1.51) | 5.06 (2.82) |
| ***Range*** | 10.0-13.0 | 3.0-11.0 | 2.0-9.0 | 1.0-1.0 | 1.0-9.0 | 1.0-6.0 | 1.0-13.0 |
| **V4 - Number of types of services** | |  |  |  |  |  |  |
| ***Mean (SD)*** | 2.2 (0.84) | 1.53 (0.69) | 1.29 (0.51) | 1.0 (0.0) | 2.0 (1.0) | 1.23 (0.43) | 1.39 (0.61) |
| ***Range*** | 1.0-3.0 | 1.0-3.0 | 1.0-3.0 | 1.0-1.0 | 1.0-3.0 | 1.0-2.0 | 1.0-3.0 |
| **V5 - Number of trained personnel groups** | | |  |  |  |  |  |
| ***Mean (SD)*** | 3.0 (0.0) | 2.58 (0.89) | 2.2 (1.05) | 0.8 (0.42) | 3.0 (0.0) | 1.73 (0.94) | 2.17 (1.04) |
| ***Range*** | 3.0-3.0 | 0.0-3.0 | 0.0-3.0 | 0.0-1.0 | 3.0-3.0 | 0.0-3.0 | 0.0-3.0 |
|  | **University** | **Central** | **Regional** | **Birth** | **Children** | **Specialty** | **All Hospitals** |
| **V6 - Rate of residents to patients** | |  |  |  |  |  |  |
| ***Mean (SD)*** | 0.52 (0.17) | 0.26 (0.12) | 0.33 (0.27) | 0.0 (0.0) | 0.39 (0.03) | 0.15 (0.26) | 0.25 (0.23) |
| ***Range*** | 0.36-0.77 | 0.0-0.51 | 0.0-1.0 | 0.0-0.0 | 0.36-0.41 | 0.0-0.77 | 0.0-1.0 |
| **V7 - Costs for research and development** | | |  |  |  |  |  |
| ***Mean (SD)*** | 1.02e8 (5.04e7) | 1.09e6 (1.87e6) | 1.49e5 (8.35e5) | 0.0 (0.0) | 1.35e7 (1.30e7) | 7.66e5 (2.08e6) | 5.16e6 (2.25e7) |
| ***Range*** | 5.98e7-1.85e8 | 0.0-6.64e6 | 0.0-5.36e6 | 0.0-0.0 | 9.32e5-2.69e7 | 0.0-8.14e6 | 0.0-1.85e8 |
| **V8 - Casemix index** | |  |  |  |  |  |  |
| ***Mean (SD)*** | 1.44 (0.12) | 0.98 (0.13) | 0.88 (0.22) | 0.38 (0.01) | 1.21 (0.23) | 1.15 (0.35) | 0.95 (0.31) |
| ***Range*** | 1.3-1.59 | 0.64-1.38 | 0.55-1.9 | 0.36-0.4 | 1.06-1.47 | 0.65-2.06 | 0.36-2.06 |
| **V9 - Ratio of rare DRGs** | |  |  |  |  |  |  |
| ***Mean (SD)*** | 0.07 (0.02) | 0.02 (0.01) | 0.01 (0.01) | 0.0 (0.0) | 0.13 (0.03) | 0.02 (0.03) | 0.02 (0.03) |
| ***Range*** | 0.05-0.09 | 0.01-0.05 | 0.0-0.04 | 0.0-0.0 | 0.11-0.16 | 0.0-0.15 | 0.0-0.16 |
| **V10 - Ratio of deliveries** | |  |  |  |  |  |  |
| ***Mean (SD)*** | 0.1 (0.03) | 0.11 (0.06) | 0.12 (0.09) | 0.99 (0.01) | 0.02 (0.01) | 0.02 (0.09) | 0.17 (0.26) |
| ***Range*** | 0.05-0.14 | 0.0-0.3 | 0.0-0.43 | 0.98-1.0 | 0.02-0.03 | 0.0-0.42 | 0.0-1.0 |

|  | **University** | **Central** | **Regional** | **Birth** | **Children** | **Specialty** | **All Hospitals** |
| --- | --- | --- | --- | --- | --- | --- | --- |
| **V11 - Ratio of children** | |  |  |  |  |  |  |
| ***Mean (SD)*** | 0.08 (0.05) | 0.06 (0.04) | 0.04 (0.03) | 0.02 (0.01) | 0.98 (0.01) | 0.03 (0.05) | 0.07 (0.15) |
| ***Range*** | 0.02-0.13 | 0.0-0.15 | 0.0-0.12 | 0.0-0.04 | 0.97-0.99 | 0.0-0.22 | 0.0-0.99 |
| **V12 - Ratio of admissions from nursing homes** | | |  |  |  |  |  |
| ***Mean (SD)*** | 0.01 (0.01) | 0.03 (0.02) | 0.02 (0.02) | 0.0 (0.0) | 0.0 (0.0) | 0.01 (0.04) | 0.02 (0.02) |
| ***Range*** | 0.01-0.03 | 0.0-0.06 | 0.0-0.08 | 0.0-0.0 | 0.0-0.0 | 0.0-0.16 | 0.0-0.16 |
| **V13 - Ratio of emergency/ ambulance admissions** | | |  |  |  |  |  |
| ***Mean (SD)*** | 0.45 (0.05) | 0.5 (0.15) | 0.5 (0.21) | 0.01 (0.01) | 0.61 (0.05) | 0.06 (0.07) | 0.38 (0.26) |
| ***Range*** | 0.39-0.51 | 0.0-0.68 | 0.01-0.89 | 0.0-0.02 | 0.55-0.65 | 0.0-0.26 | 0.0-0.89 |
|  | | |  |  |  |  |  |
| **V14 - Ratio of admissions during weekend/ night** | | |  |  |  |  |  |
| ***Mean (SD)*** | 0.2 (0.02) | 0.19 (0.06) | 0.19 (0.08) | 0.01 (0.0) | 0.31 (0.02) | 0.03 (0.06) | 0.15 (0.1) |
| ***Range*** | 0.17-0.22 | 0.01-0.27 | 0.0-0.36 | 0.0-0.01 | 0.29-0.32 | 0.0-0.29 | 0.0-0.36 |
| **V15 - Ratio of admissions from outside of canton** | | |  |  |  |  |  |
| ***Mean (SD)*** | 0.24 (0.17) | 0.12 (0.1) | 0.21 (0.2) | 0.3 (0.23) | 0.42 (0.22) | 0.31 (0.25) | 0.22 (0.2) |
| ***Range*** | 0.07-0.51 | 0.02-0.48 | 0.02-1.0 | 0.04-0.84 | 0.24-0.67 | 0.02-0.87 | 0.02-1.0 |

|  | **University** | **Central** | **Regional** | **Birth** | **Children** | **Specialty** | **All Hospitals** |
| --- | --- | --- | --- | --- | --- | --- | --- |
| **V16 - Rate of DRGs to patients** | |  |  |  |  |  |  |
| ***Mean (SD)*** | 0.02 (0.0) | 0.04 (0.01) | 0.1 (0.06) | 0.03 (0.02) | 0.08 (0.02) | 0.06 (0.03) | 0.06 (0.05) |
| ***Range*** | 0.02-0.02 | 0.02-0.06 | 0.03-0.3 | 0.01-0.05 | 0.06-0.09 | 0.02-0.12 | 0.01-0.3 |
| **V17 - Rate of specialized services to patients** | | |  |  |  |  |  |
| ***Mean (SD)*** | 0.02 (0.0) | 0.04 (0.02) | 0.11 (0.12) | 0.0 (0.0) | 0.09 (0.02) | 0.1 (0.21) | 0.07 (0.12) |
| ***Range*** | 0.02-0.02 | 0.0-0.07 | 0.0-0.75 | 0.0-0.0 | 0.07-0.11 | 0.0-1.0 | 0.0-1.0 |
| **V18 - Expected loss potential based on DRG mix** | | |  |  |  |  |  |
| ***Mean (SD)*** | 342.06 (80.88) | 103.06 (31.37) | 66.92 (34.57) | 4.63 (1.39) | 400.42 (30.25) | 57.6 (71.48) | 91.47 (89.74) |
| ***Range*** | 252.54-462.52 | 35.65-193.36 | 17.57-199.16 | 1.99-6.59 | 380.54-435.22 | 7.53-329.52 | 1.99-462.52 |
| **V19 - Location in large agglomeration** | | |  |  |  |  |  |
| ***Mean (SD)*** | 0.67 (0.17) | 0.23 (0.3) | 0.1 (0.22) | 0.24 (0.36) | 0.54 (0.47) | 0.39 (0.38) | 0.24 (0.33) |
| ***Range*** | 0.52-0.91 | 0.0-0.91 | 0.0-0.91 | 0.0-0.85 | 0.0-0.85 | 0.0-0.91 | 0.0-0.91 |
| **V20 - Location in medium-sized/ small agglomeration** | | | |  |  |  |  |
| ***Mean (SD)*** | 0.0 (0.0) | 0.18 (0.17) | 0.19 (0.18) | 0.14 (0.13) | 0.12 (0.21) | 0.14 (0.19) | 0.17 (0.17) |
| ***Range*** | 0.0-0.01 | 0.0-0.58 | 0.0-0.58 | 0.0-0.37 | 0.0-0.37 | 0.0-0.53 | 0.0-0.58 |

|  | **University** | **Central** | **Regional** | **Birth** | **Children** | **Specialty** | **All Hospitals** |
| --- | --- | --- | --- | --- | --- | --- | --- |
| **V21 - Location in peri-urban/ rural area** | | |  |  |  |  |  |
| ***Mean (SD)*** | 0.33 (0.17) | 0.59 (0.21) | 0.71 (0.22) | 0.62 (0.28) | 0.34 (0.26) | 0.47 (0.26) | 0.59 (0.25) |
| ***Range*** | 0.09-0.48 | 0.09-0.93 | 0.09-1.0 | 0.15-0.87 | 0.15-0.63 | 0.09-0.88 | 0.09-1.0 |
| **V22 - Median income of patients** | |  |  |  |  |  |  |
| ***Mean (SD)*** | 65.94 (6.89) | 65.48 (12.7) | 64.77 (12.15) | 62.64 (3.72) | 66.63 (6.83) | 65.78 (7.97) | 65.1 (10.76) |
| ***Range*** | 56.29-73.52 | 48.16-117.47 | 51.38-98.48 | 56.6-67.24 | 60.29-73.86 | 52.23-87.27 | 48.16-117.47 |
| **V23 - Healthcare density** | |  |  |  |  |  |  |
| ***Mean (SD)*** | 0.24 (0.85) | 0.22 (1.09) | -0.4 (0.76) | 0.14 (0.97) | 0.48 (0.99) | 0.12 (0.93) | -0.01 (0.96) |
| ***Range*** | -0.35-1.7 | -1.14-2.53 | -1.33-1.8 | -0.76-2.42 | -0.35-1.58 | -1.11-2.23 | -1.33-2.53 |

*Note.* Variables V6 and V17 were rescaled to make their values readable with the chosen scale. University = university hospitals, Central = central general hospitals, Regional = regional general hospitals, Birth = birth centers, Children = children’s hospitals, Specialty = specialty hospitals.
